# Bismuth Vanadium Oxide Can Promote Growth and Activity in Arabidopsis thaliana

**DOI:** 10.3389/fchem.2021.766078

**Published:** 2021-11-11

**Authors:** Cong Gao, Shuai Lu, Yongzhou Wang, Hao Xu, Xiaoxiao Gao, Yiwen Gu, Hongyun Xuan, Baohua Wang, Huihua Yuan, Yunying Cao

**Affiliations:** School of Life Sciences, Nantong University, Nantong, China

**Keywords:** bismuth vanadium, *Arabidopsis thaliana*, roots, reactive oxygen generation, gene expression, bacteriostasis

## Abstract

The excellent properties of nanomaterials have been confirmed in many fields, but their effects on plants are still unclear. In this study, different concentrations of bismuth vanadate (BV) were added to the growth medium to analyze the growth of seedlings, including taproots, lateral roots, leaf stomata, root activity, and superoxide anion O_2_^.-^ generation. Gene expression levels related to root growth were determined by quantitative PCR in *Arabidopsis thaliana*. The results showed that BV promoted the growth of taproots and the development of lateral roots, enhanced the length of the extension zone in roots, increased the number and size of leaf stomata and root activity, reduced the accumulation of ROS in seedlings, and changed the expression levels of genes related to polyamines or hormones. At the same time, we investigated the antibacterial activity of BV against a variety of common pathogens causing crop diseases. The results showed that BV could effectively inhibit the growth of Fusarium wilt of cotton and rice sheath blight. These results provide a new prospect for the development of nanomaterial-assisted plants, which is expected to become one of the ways to solve the problem of controlling and promoting the development of plants. At the same time, it also provides a reference for the study of the effect of BV on plants.

## Introduction

Compared with traditional materials, nanomaterials have many advantages and are applied in the information industry, environmental industry, energy (Zhao et al., 2018; Hao et al., 2021; Zhao et al., 2021) and environmental protection, biological medicine, and other fields (Castiglione et al., 2011). The growth and development of plants are regulated by many factors, such as temperature and hormones (Brandhoff et al., 2017; Ibañez et al., 2017; Kim et al., 2017; Sun et al., 2019; Gómez-Merino et al., 2020). An increasing number of studies have shown that nanomaterials also have an impact on the growth and development of plants (Thuesombat et al., 2014). For example, modified polystyrene nanomaterials with different polarities could inhibit root development in *Arabidopsis* (Sun et al., 2020). Copper oxide nanoparticles (CuONPs) regulated the phenotype of mung bean (Nair et al., 2014). CuONPs can gradually decrease the mitotic index of onion root tips gradually and increase the abnormal index (Nagaonkar et al., 2015). Furthermore, the effects of nano-TiO_2_ foliage intervention on cadmium bioaccumulation, stress kinase, and potential dietary health risks in cowpea plants were also reported (Ogunkunle et al., 2020). Moreover, the degree of lignification of the xylem in roots and stems of fenugreek treated with nanosilicon materials was significantly higher than that of control plants (Nazaralian et al., 2017). Most of the nanomaterials mentioned above showed toxic effects on plants; however, some of them demonstrated a positive role. A study reported that graphene oxide promoted the growth of watermelon, including increasing root length, leaf area, leaf number, and flower bud formation (Park et al., 2020). Multiwalled carbon nanotubes (MWCNTs) could stimulate the seed germination of three important crops (barley, soybean, and maize) and enhance the root length in *Phaseolus mungo* seedlings and the germination index of *Brassica juncea* at low concentrations (Ghodake et al., 2010; Mondal et al., 2011; Lahiani et al., 2013). However, their application in plant-related fields is still limited, and due to the variety and different characteristics of nanomaterials, different materials show different effects on plants.

Metal vanadates have been widely used in applications such as batteries, implantable cardiac defibrillators, and photocatalysts (Sivakumar et al., 2015). Specifically, bismuth vanadate nanomaterials (herein referred to as BVs) have emerged as promising candidates due to their unique nontoxicity, chemical stability, optical properties, and ferro-elastic properties (Sarkar et al., 2012). Various applications of BV have been well-studied in eco-friendly yellow pigments, water splitting processes, sensors, and pollutant degradation. Recently, BV has garnered notable attention in biological applications. A BV composite material exhibited excellent potential for the inactivation of *Escherichia coli* (Sharma et al., 2016; Guan et al., 2018; Regmi et al., 2018). This study provided evidence about the positive effects of BV nanomaterials on the microbiome. However, currently, no or few attempts have been made to explore the effect of BV nanomaterials on plants. Therefore, the objective of this study was to use *Arabidopsis thaliana* as a model plant to determine the effect of BV on some factors, including the length of taproots, the number of lateral roots, the number and size of stomata in leaves, the activity of plant roots, superoxide anion O_2_^.-^ generation and the accumulation of BV in seedlings, antimicrobial activity, and the expression of genes related to roots. This evaluation was based on determining the effects of applying different concentrations of BV on seedlings to clarify the effect of BV on *Arabidopsis thaliana*.

## Materials and Methods

### Plant Material

Seeds of *Arabidopsis thaliana* Columbia-0 (Col-0) were surface-sterilized with 70% ethanol and 20% bleach. Materials were grown at 22°C/20°C under a photoperiod with 16 h of light and 8 h of dark and a light intensity of 100 μmol m^−2^ s^−1^ in an incubator (QY-14; Nanjing Quanyou Electronic Technology Co., Ltd, China).

### Preparation and Characterization of BV

Bi(NO_3_)_3_⋅5H_2_O (2.1830 g) and EDTA (4 g) were dissolved in dilute HNO_3_ solution (50 ml, 2 mmol L^−1^) and stirred at 90°C for 30 min (200 r min^−1^) to obtain solution A. NH_4_VO_3_ (0.5260 g) was dissolved in deionized water (50 ml) at 60°C to obtain solution B. Then, solution B was mixed with solution A, and the pH was adjusted to 7 by adding NH_4_OH. The abovementioned mixed solution was stirred for 1 h at 50°C, poured into a 150-ml Teflon-lined stainless-steel autoclave, and maintained at 180°C for 6 h. The prepared precipitate was washed with ethanol and deionized water several times and vacuum-dried at 75°C overnight to form BV nanomaterials. Sample morphology and surface elemental composition were examined by scanning electron microscopy (SEM; JSM6510, JEOL, Japan) coupled with energy-dispersive spectrometry (EDS) at an accelerating voltage of 10 kV. X-ray diffraction (XRD; Ultima IV, Rigaku, Japan) patterns of the samples were recorded by using high-intensity Cu Ka radiation (λ = 0.154 nm) in the range of 2θ = 10°–80° at a scan rate of 5° min^−1^.

### Preparation of Roots for Analysis

With respect to plants grown in solid media, *Arabidopsis thaliana* seeds were germinated on a square plate (10 cm × 10 cm) that contained sterilized solidified half-strength MS (Sigma-Aldrich, St. Louis, MO, United States) media consisting of 0.8% agar (Affymetrix, Inc. Cleveland, Ohio, United States) and 1% sucrose. Ten grams of BV powder was dissolved in 1 L of deionized water and sterilized in an autoclave (SANYO Labo Autoclave, MLS-3020). The BV solution that had been sonicated for 1 h and was blended with a sterilized half-strength MS medium by stirring with 0, 20, 50, 100, and 200 μg ml^−1^ nanomaterials and the resulting medium (hereafter referred to as the BV/MS medium). In total, 30–40 seeds were planted in the BV/MS medium, placed at 4°C for 2 days, and then transferred to a growth chamber as described above. The roots were imaged using a scanner, and the primary root length was measured using ImageJ software (National Institutes of Health, United States) after 6 days of exposure. The lateral root number was analyzed after 11 days of exposure. The roots of the plants exposed to nanomaterials for 6 days were stained with propidium iodide for viability testing of the meristem, extension zone length, tip diameter, and rootcap size of the primary root as described previously (Napsucialy-Mendivil et al., 2014).

### Measurement of BV Content

Six-day-old control and BV-treated *Arabidopsis thaliana* were divided into roots and leaves, dried (105°C for half an hour and 80°C for 3 days), and ground into powder. Subsequently, the powder was used to measure the BV content using XRD.

### Measurement of the Number and Size of Stomata

Six-day-old control and BV-treated *Arabidopsis thaliana* were dehydrated with different concentrations of ethanol (30, 50, 70, 80, and 90% for 20 min and 100% for 40 min, repeated three times), dried by using a critical point dryer (EM CPD 300, Leica, Germany), and coated with a film with ion sputtering equipment (EM ACE 600, Leica, Germany). The SEM (JSM6510, JEOL, Japan) was used to take photos of dehydrated materials, and the photos were imported into ImageJ to count the number and size of stomata. The calculation formula of stomatal density (SD) was as follows: SD = N/S, where N is the number of stomata in the visual field and S is the area of the visual field. The formula for calculating the stomatal size (SS) was SS = L (length)*W (width)*3.14/4.

### Detection of Superoxide Anion Radical O_2_^.-^ and Root Activity

To visualize O_2_^.-^ and root activity in plants *in situ*, nitroblue tetrazolium (NBT) and 2, 3, 5-triphenyl tetrazolium chloride (TTC) staining was performed, respectively, as described previously (Kong et al., 2018; Tanaka et al., 2020) and modified slightly. O_2_^.-^ generated in seedlings was measured by incubating the plants in 1% NBT within 20 mmol L^−1^ potassium phosphate, washing with distilled water, and decolorizing with 70% ethanol solution in water at 90°C for 20 min. The root activity was measured by incubating the plants in 2% TTC at 37°C for 5 h. Images of the plants or roots were obtained under brightfield illumination.

### Antimicrobial Effect of Nanomaterials

To clarify the antimicrobial effect of nanomaterials, we selected two common pathogens that cause crop diseases, namely, *Thanatephorus cucumeris* (Frank) Donk, which causes rice diseases, and *Fusarium oxysporum* f. sp. *vasinfectum* causing cotton diseases. The activated bacteria were added to the LB liquid medium by adding 200 μg ml^−1^ nanomaterials. The OD600 value was measured after 12 h of incubation at 28°C. After centrifugation, the bacterial fluid was fixed with 2.5% glutaraldehyde, dehydrated with gradient ethanol, and vacuum-dried (DZF-6020, Yihen, China). Finally, the morphology of bacteria was observed by using a SEM (JSM6510, JEOL, Japan).

### RT–qPCR Analysis

Plants were grown on half-strength MS media with either BV (200 μg ml^−1^) or without nanomaterials. Approximately, 6-day-old primary roots and 11-day-old roots were harvested. *ADC-1* (*AT2G16500*), *DAR-2 (AT2G39830),* and *IQM3 (AT3G52870)* were selected to analyze the expression of the taproot, while *ARF19 (AT1G19220)*, *CKX1 (AT2G41510)*, *ERF6 (AT4G17490)*, and *IQM3* (*AT3G52870*) were selected to analyze the expression of the lateral root. Total RNA was isolated using TRIzol reagent (Invitrogen) and converted to complementary DNA (cDNA) using a Transcriptor First Strand cDNA Synthesis Kit (Roche) following the manufacturer’s protocol. qPCR was performed using a 7500 Real-time PCR Detection System (Bio–Rad) in conjunction with the Fast Start universal SYBR Green Master Mix (Roche). *ACT2* (*AT3G18780*) was used as a reference gene to normalize the data, and the relative expression levels were calculated using the 2^−ΔΔCT^ method, as described previously (Cao et al., 2013). The primers used for qPCR are listed in Supplementary Table S1.

### Statistical Analyses

Without special instructions, all experiments were repeated at least three times. SPSS 20.0 and SigmaPlot 10.0 were used for statistical analysis and plotting. Statistical differences were analyzed by Duncan’s test. The data were considered significant in accordance with the following criteria (*p* < 0.05).

## Results

### Fabrication and Characterization of Nanomaterial

**FIGURE 1:**
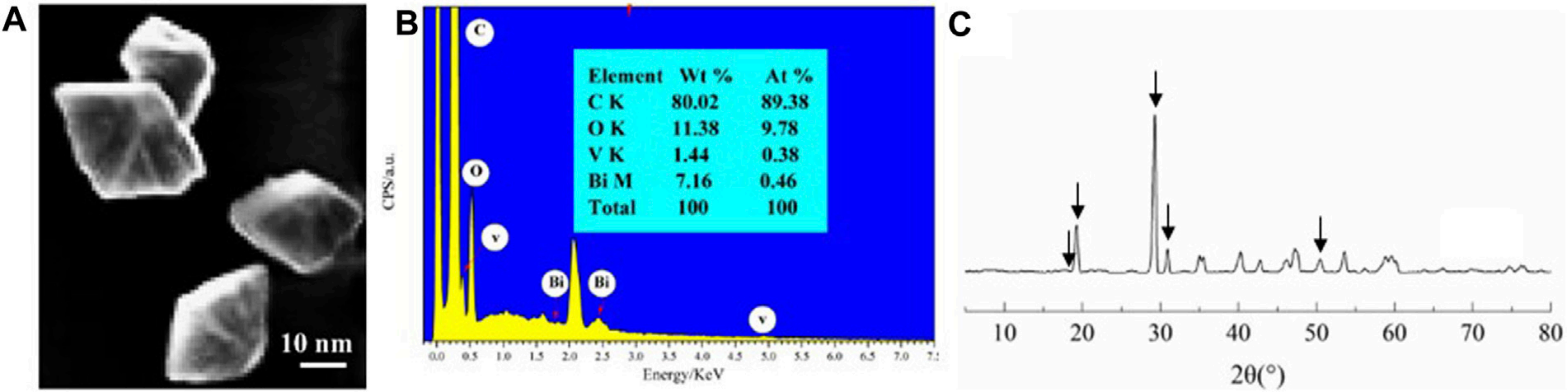
Characterization of BV nanomaterial SEM **(A)**, EDS spectra **(B)**, and XRD patterns **(C)** images of BV. Arrows indicate the characteristic peak of BV.

The morphology and structures of the as-prepared BV nanomaterials were detected by SEM (Figure 1). Geometrically shaped–like nanoparticles were observed for BV, and their average diameter was approximately 16 ± 3 nm (Figure 1A). In addition, C, O, Bi, and V were clearly displayed in the EDS spectra (Figure 1B), confirming the presence of BV nanomaterials. The phase purities and crystallinities of the BV nanomaterials were further characterized by XRD analysis (Figure 1C). Obviously, the diffraction peaks at 2θ = 18.9°, 19.3°, 29.3°, 30.9°, and 53.6° revealed the (110), (011), (121), (040), and (161) planes for BV nanomaterials in the XRD pattern, respectively, indexed to monoclinic scheelite-type BV (JCPDS no. 14–0688).

### Effects of Nanomaterial on *Arabidopsis* Roots

**FIGURE 2:**
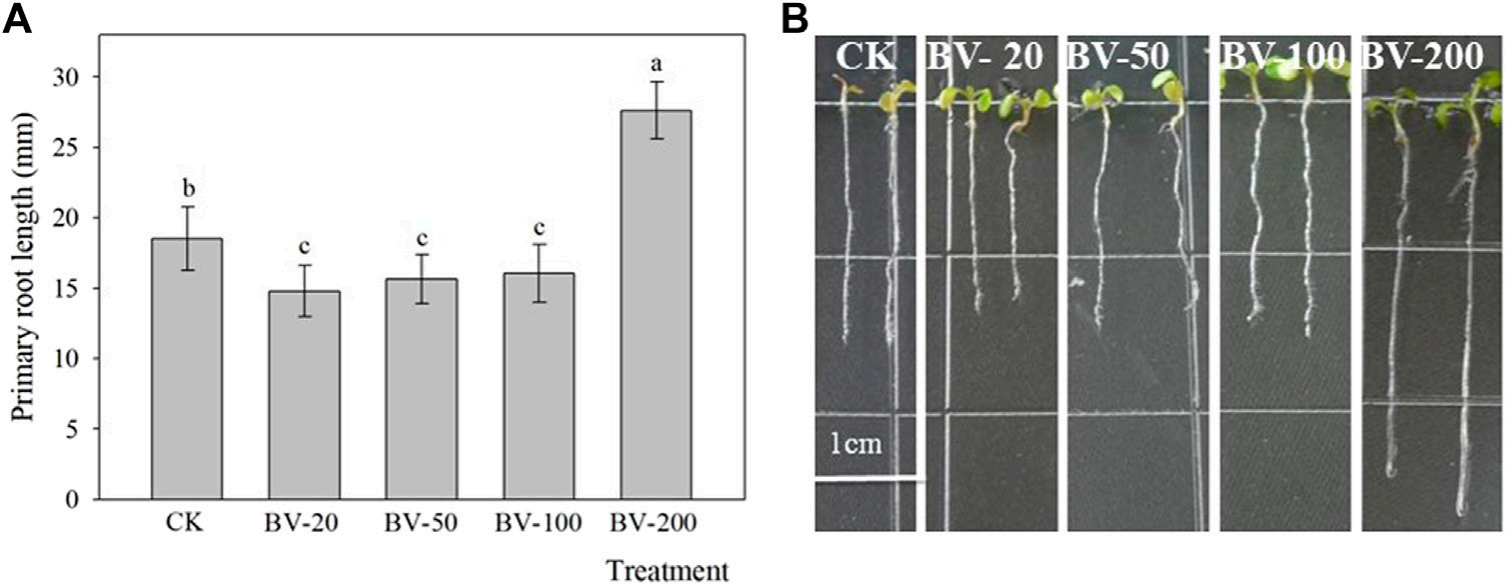
Effects of different concentrations of BV nanomaterial on the primary root length of *Arabidopsis.* Bar of **(A)** was standard error. Scale bar = 1 cm. CK, BV-20, BV-50, BV-100, and BV-200 of **(A and B)** were added to the MS medium with 0, 20, 50, 100, and 200 μg ml^−1^ of BV, respectively. N = 30. Different lowercase letters above the bar of **(A)** indicate that there were significant differences among the treatments at *p <* 0.05.

To confirm the influence of BV on the primary root length and lateral root numbers, these two traits were evaluated after 6 and 11 days of plant growth on the various BV/MS media. Figure 2 shows that the nanomaterial affects the length of primary roots in *Arabidopsis*, and different concentrations of nanomaterials demonstrated inconsistent changes. Compared with the control, 20, 50, and 100 μg ml^−1^ BV significantly reduced the length of taproots by approximately 13.0～20.0%, while 200 μg ml^−1^ BV remarkably enhanced the length by approximately 49.0%. This may imply that BV has a dual effect on the length of plant roots.

**FIGURE 3:**
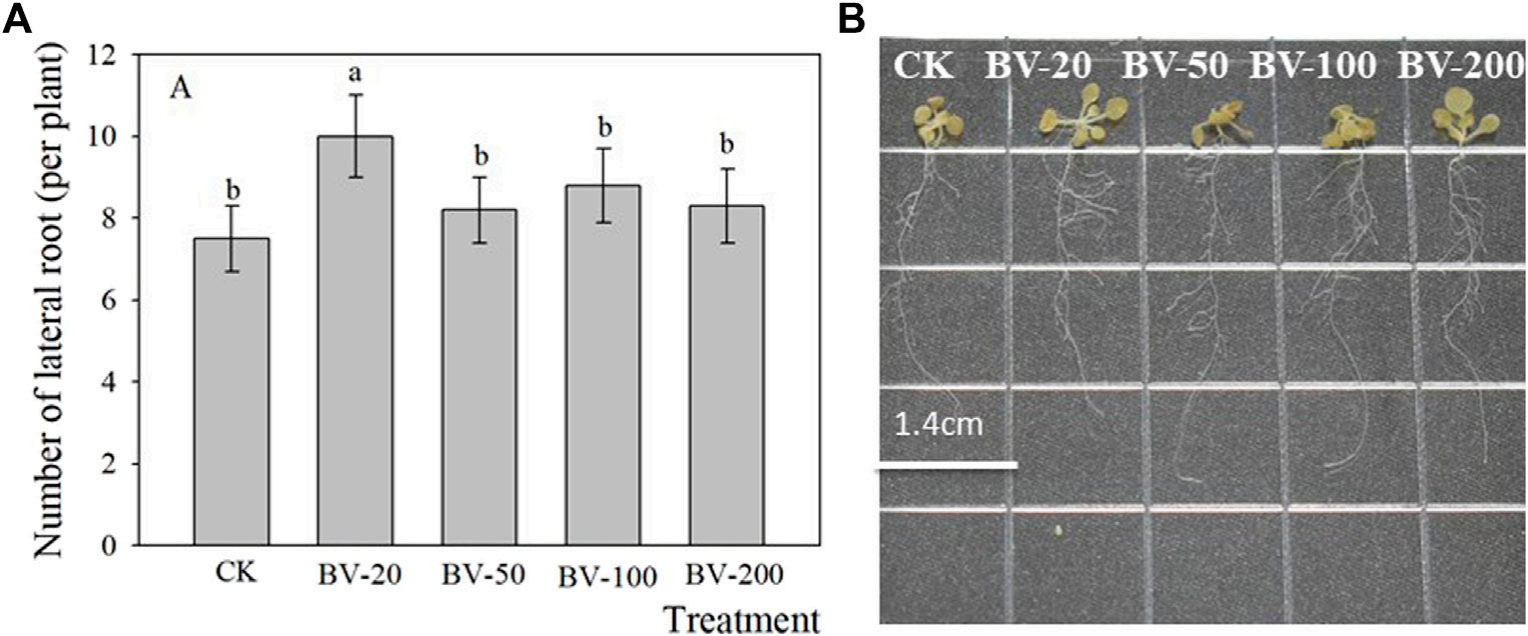
Effects of different concentrations of BV nanomaterials on the number of lateral roots in *Arabidopsis.* Bar of **(A)** was standard error. CK, BV-20, BV-50, BV-100, and BV-200 of **(A and B)** were added to the MS medium with 0, 20, 50, 100, and 200 μg ml^−1^ of BV, respectively. N = 15. Different lowercase letters above the bar of **(A)** indicate that there were significant differences among the treatments at *p <* 0.05.

In addition, we also observed that the addition of BV nanomaterials can also affect the lateral root number (Figure 3). BV treatment at different concentrations increased the number of lateral roots of all the plants, and the difference in the 20-μg ml^−1^ treatment was significant. There was no significant difference among the other treatments compared to the treatment without the addition of the nanomaterial. In general, the effect of low concentration was more beneficial to increase the number of lateral roots.

**FIGURE 4:**
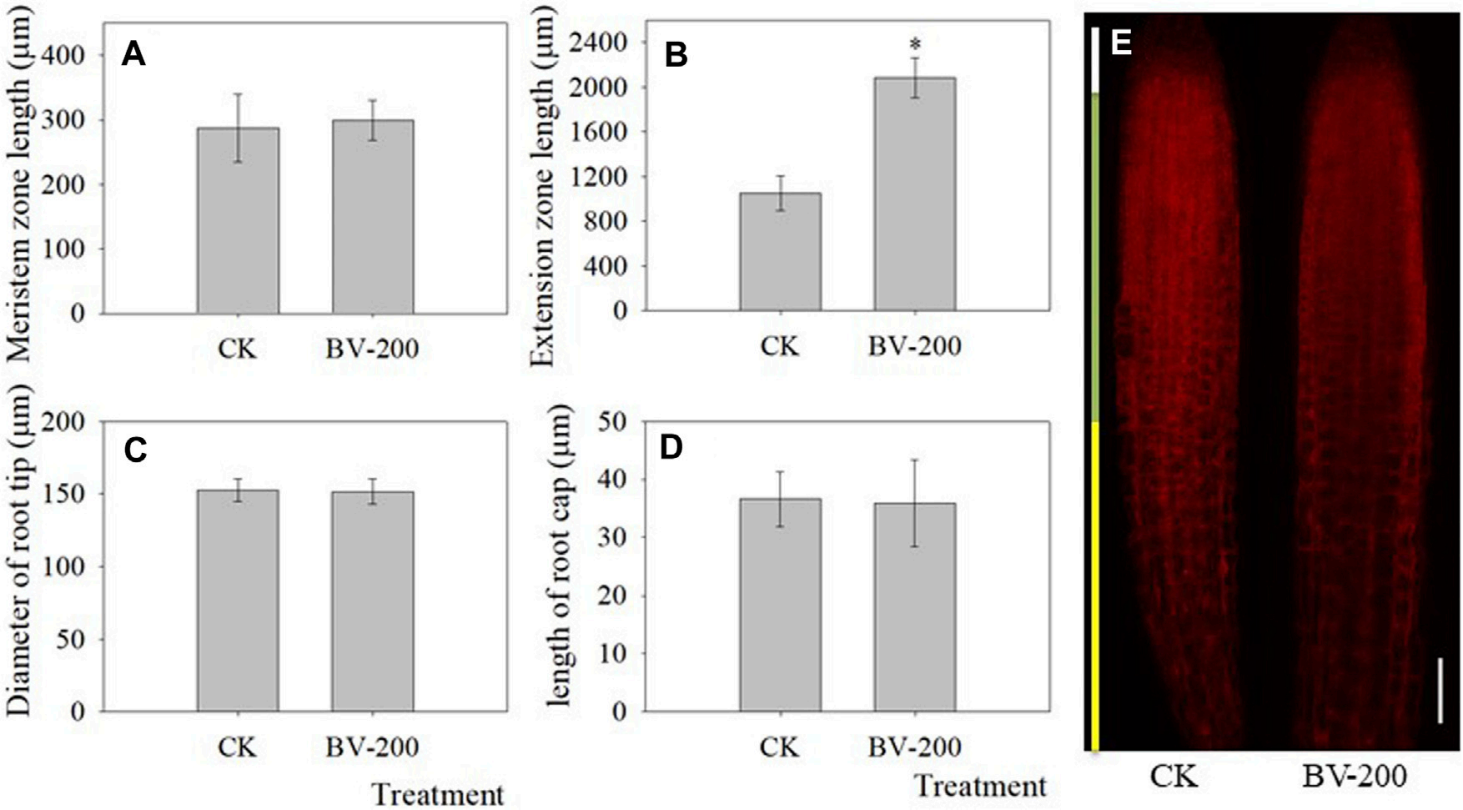
Effect of BV nanomaterials on primary root length in *Arabidopsis*. **(A)** Meristem zone length of primary root. **(B)** Extension zone length of primary root. **(C)** Diameter of root tip. **(D)** Length of the taproot cap. **(E)** Representative images of roots stained with PI to outline the living cells, showing the root cap, meristem domain, and extension zone after 4 days of incubation. Scale bar = 50 μm. CK: the control without adding BV. BV-200: 200 μg ml^−1^ BV treatment. N = 30. Asterisks above the bar indicate that there were significant differences between the two treatments at *p* < 0.05.

Based on the fact that BV promoted the development of taproots, the influence of BV nanomaterials was further explored by measuring different positions of roots (Figure 4). BV treatment enhanced the length of the extension zone, but there was little effect on that of the meristem zone and taproot cap. Therefore, the results suggested that BV might have an effect on the extension zone and leads to an increase in the length of the taproot. Furthermore, the diameter of the primary root was also analyzed. Unfortunately, there was no significant change in the diameter, suggesting that BV has a limited effect on root diameter.

### The Number and Size of Stomata Increased After BV Treatment

**FIGURE 5:**
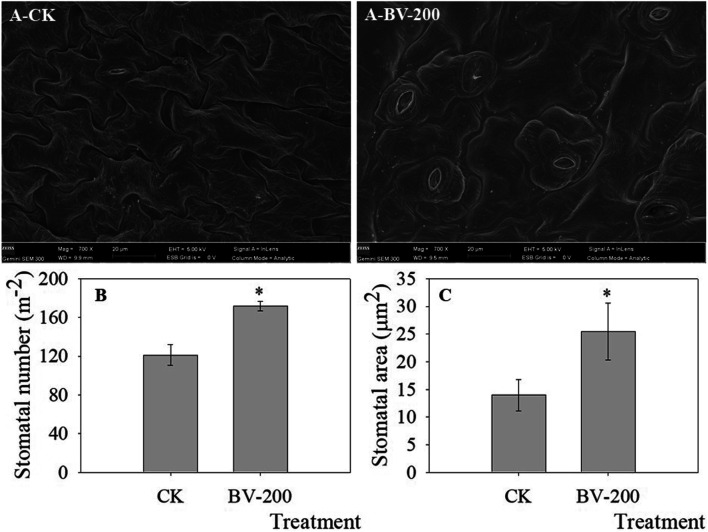
Stomatal density and size in true leaves revealed by SEM. A-CK and A-BV-200 were the control and 200 μg ml^-1^ BV, respectively. Bars in Figure A were 20 µm (mag = 700 × ). **(A)** Stomatal number per square (stomatal density). **(B)** Mean area per stomata (stomatal size, µm^2^).   Asterisks above the bar indicate that there were significant differences between the two treatments at *p* < 0.05.

Since the roots were affected after BV treatment, we decided to observe whether the leaves were also affected. As shown in Figure 5, we found that the number and size of stomata increased significantly. After BV treatment, the number of stomata reached 171, while the number of stomata in the control group was 121. The stomatal size also showed a similar trend. This indicated that an appropriate amount of BV could promote stomatal development. Interestingly, compared with the control, the area of cells around the stomata was also larger after BV treatment.

### BV was Mainly Concentrated in Leaves Rather Than Roots

**FIGURE 6:**
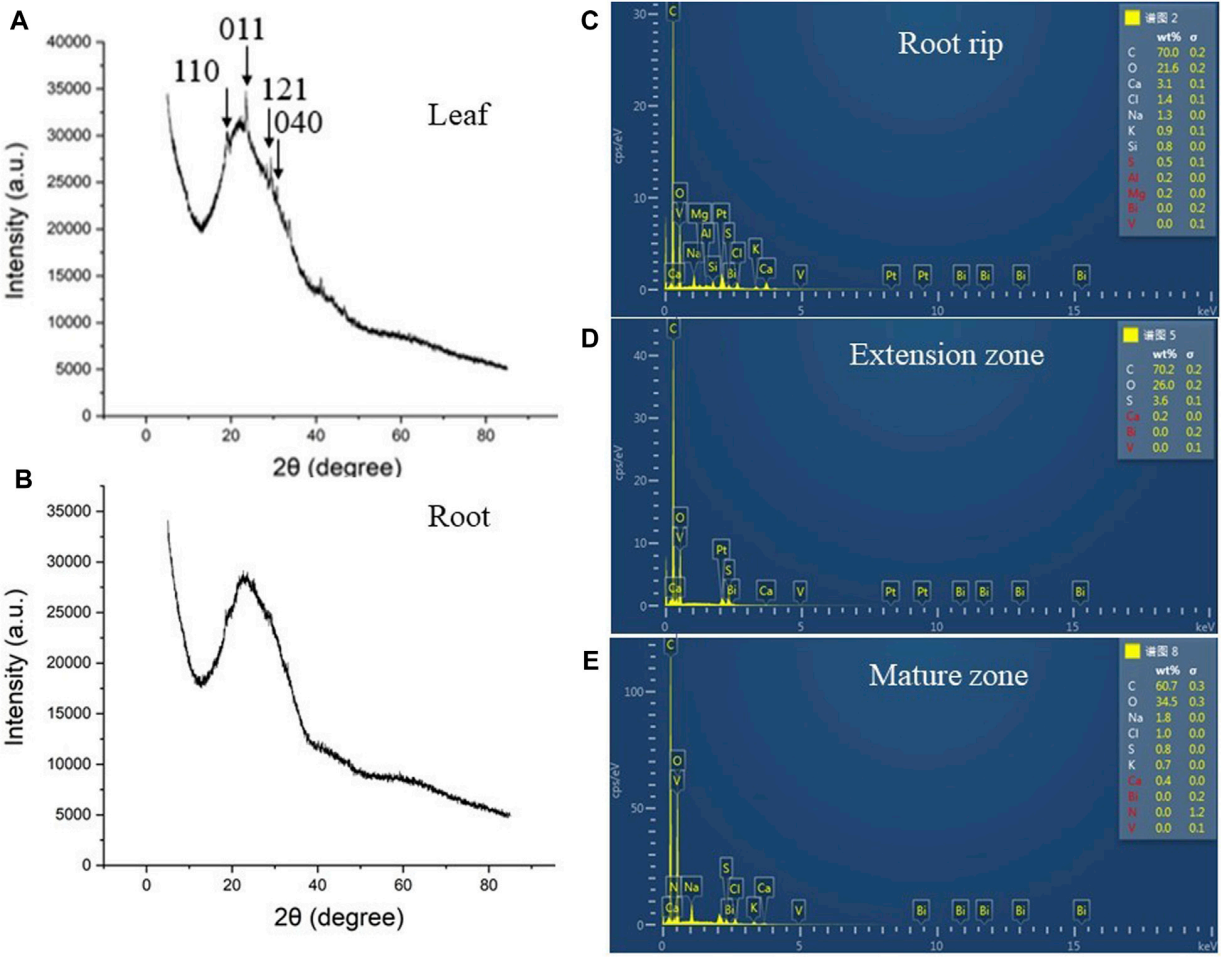
XRD analysis of leaves and taproots and ESD analysis of taproots. **(A)**: XRD analysis of leaves in 200 μg ml^−1^ BV treatment. **(B)**: XRD analysis of taproots in 200 μg ml^−1^ BV treatment. **(C)**: ESD analysis of root tips in taproots in 200 μg ml^−1^ BV treatment. **(D)**: ESD analysis of the extension zone in taproots in 200 μg ml^−1^ BV treatment. **(E)**: ESD analysis of the mature zone in taproots in 200 μg ml^−1^ BV treatment. The arrow indicates the characteristic peak of BV. N = 3.

Considering that the roots and leaves were affected, we determined the distribution of BV. As shown in the figure, leaf XRD diagrams of BV treatment clearly show the (110) (2θ = 18.9°), (011) (2θ = 19.3°), (121) (2θ = 29.3°), and (040) (2θ = 30.9°) characteristics of the crystal peak (Figure 6A). However, the characteristic plane peaks of BV can hardly be seen in the XRD patterns of the roots (Figure 6B). ESD results also showed that there was no accumulation of BV in roots (Supplementary Figure S1), including the root rip, extension zone, and mature zone (Figures 6C–E), indicating that BV nanomaterials were mostly distributed in the leaves but had low content in the roots.

### The Root Activity and O_2_^.−^ Production Analysis

**FIGURE 7:**
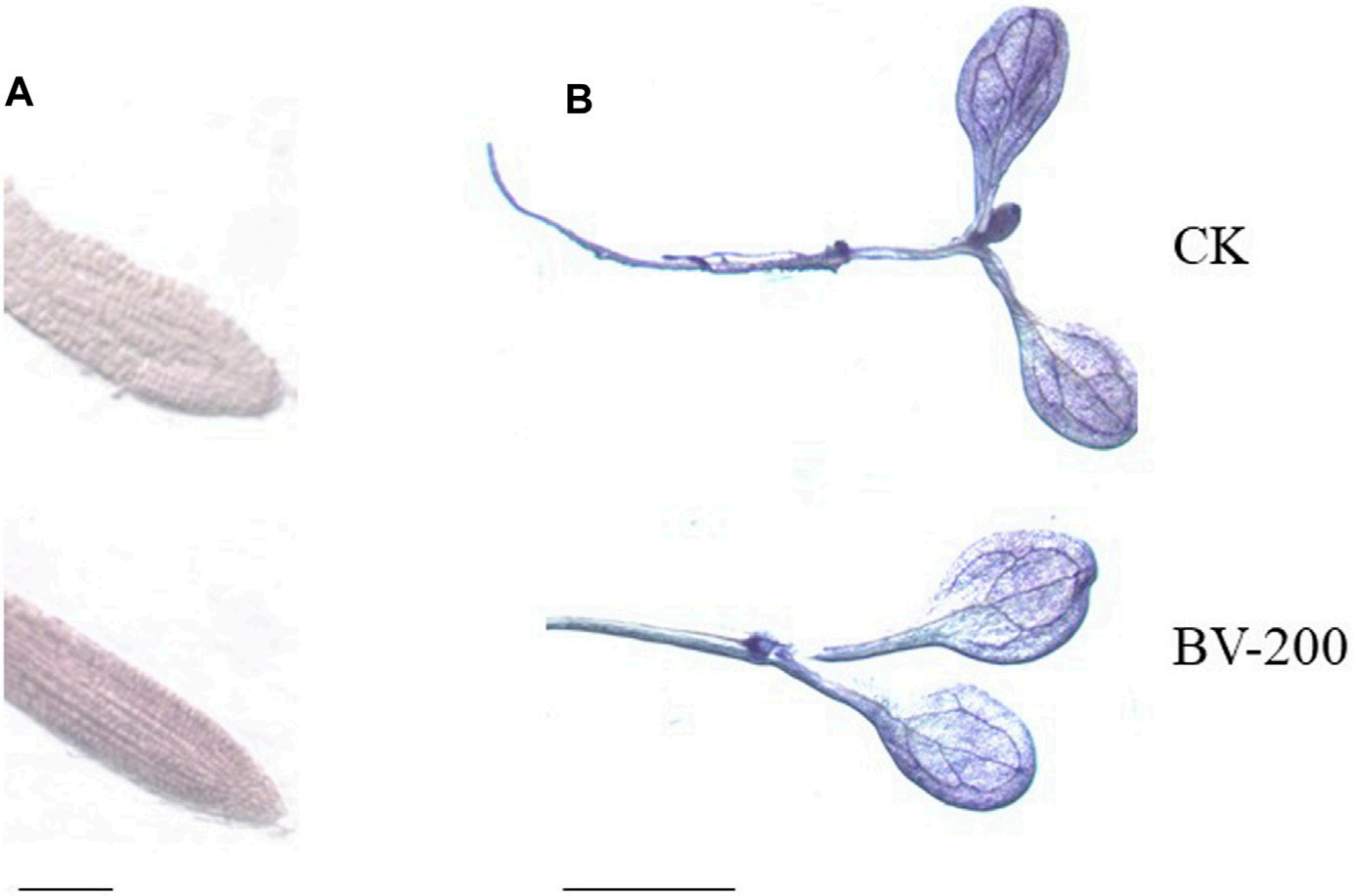
Effect of BV nanomaterial treatment on *Arabidopsis* tissue staining. **(A)** TTC staining, scale bar = 5 mm. **(B)** NBT staining, scale bar = 2 mm. CK, the control without BV nanomaterials. BV-200, 200 μg ml^−1^ BV treatment. N = 6.

The abovementioned experiments showed that BV promoted root and leaf development in plants. Therefore, we tried to clarify whether the root activity and O_2_^.−^ of seedlings also changed. O_2_^.−^ belongs to the ROS group, which has strong oxidizability. It plays an important role in the physical reaction process (Wang et al., 2007). Consequently, TTC and NBT staining were performed (Figure 7). After growing in the half-strength MS medium for 6 days, we found that the root tip color of 200 μg ml^−1^ BV was significantly darker than that of the control with TTC staining, indicating that the seedlings treated with appropriate concentrations of nanomaterials increased the root activity of *Arabidopsis* (Figure 7A). In addition, NBT staining of the leaves showed that the color of the control group was significantly darker than that of BV treatment (Figure 7B). This also indicated that BV treatment could reduce the production of ROS O_2_^−^ in seedlings.

### Antimicrobial Effect of Nanomaterials

**FIGURE 8:**
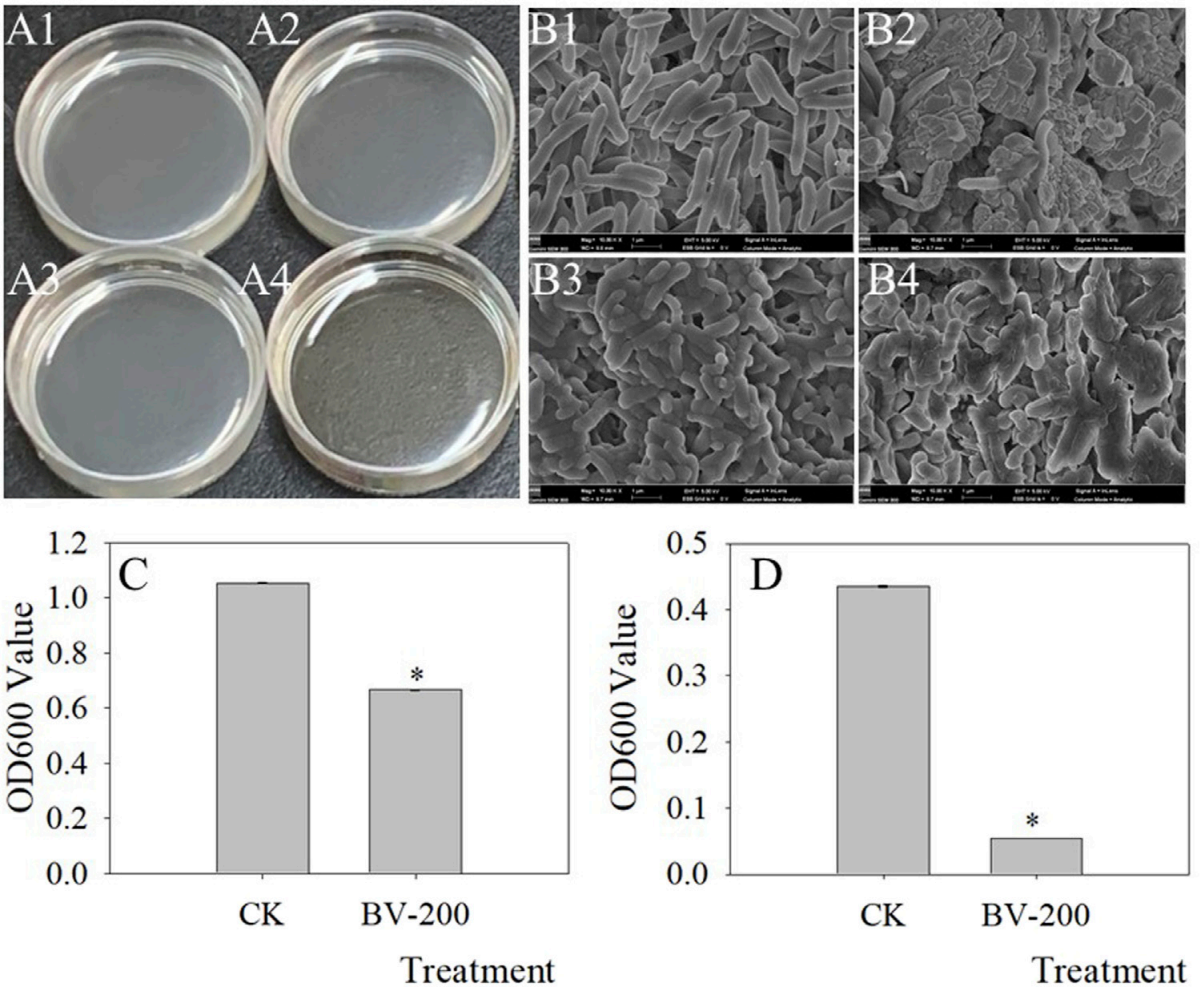
Antimicrobial effect of BV nanomaterials. Images of bacterial fluid **(A)**, SEM **(B)** of bacteria, and OD600 value of bacterial fluid **(C,D)**. A1, A3, B1, and B3 were the controls only with added bacteria. A2, A4, B2, and B4 were the treatments with added bacteria and 200 μg ml^−1^ BV. A1, A2, B1, and B2 were added to *Fusarium oxysporum* f. sp. *vasinfectum.* A3, A4, B3, and B4 were added to *Thanatephorus cucumeris* (Frank) Donk*.* Bar of C and D was standard error. Asterisks above the bar of C and D indicate that there were significant differences between the treatments at *p <* 0.05. N = 3.

Given the reported antimicrobial activity of BV (Xiang et al., 2019), antimicrobial experiments with BV were performed (Figure 8). We selected two common pathogens that caused crop diseases and found that BV had a significant inhibitory effect on them. *Fusarium oxysporum* f. sp. *vasinfectum* can cause Fusarium wilt of cotton, and *Thanatephorus cucumeris* (Frank) Donk can cause sheath blight of rice. As shown in the figure, the OD600 of these two bacteria decreased significantly in the medium supplemented with BV after 12 h of culture (Figures 8C,D). In particular, the pathogen of rice sheath blight can be observed directly according to the phenotype. The medium with BV is very limpid (Figure 8A3), while the medium without BV is very turbid (Figure 8A4). Furthermore, we found that the morphology of both the bacteria changed after adding BV as determined by SEM analysis (Figures 8B2,B4). All of these results showed that BV had antibacterial activity.

### Changes in Gene Expression Related to Root Development in *Arabidopsis*

**FIGURE 9:**
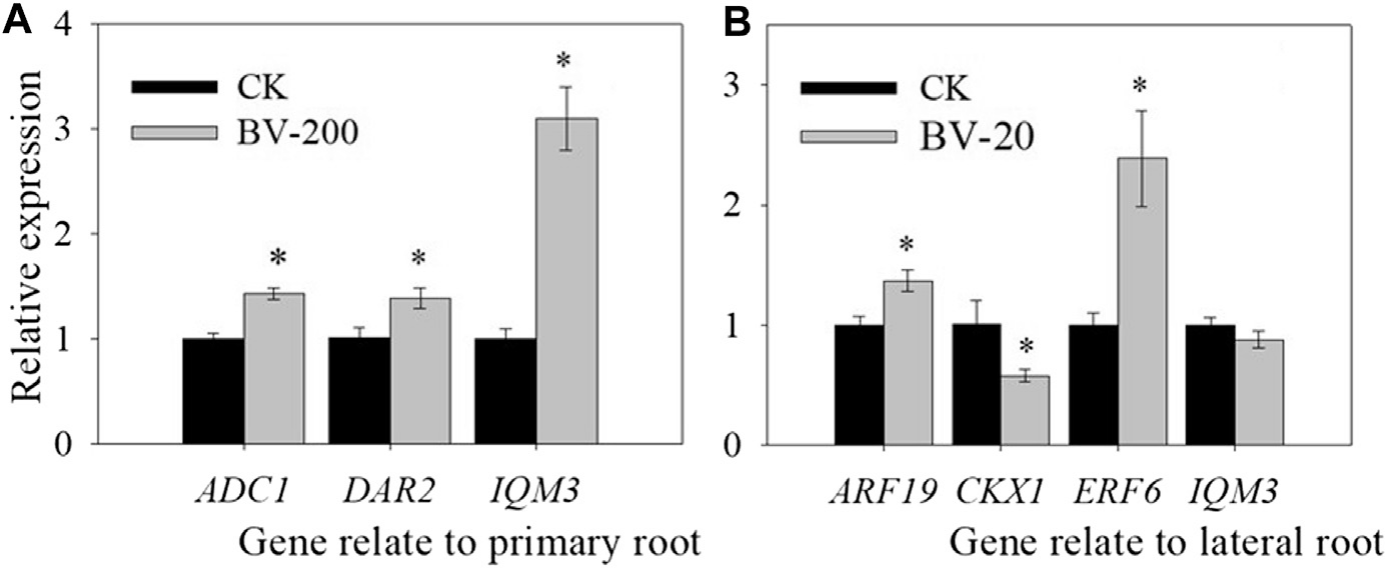
Effect of nanomaterial BV on relative gene expression of primary root length and lateral root numbers in *Arabidopsis*. **(A)** Relative gene expression of primary root length. **(B)** Relative gene expression of lateral root number. CK: Without nanomaterial treatment. 20 and 200 were treated with BV about 20 μg ml^−1^ and 200 μg ml^−1^. N = 3. Error of figure was standard error. Asterisks above the bar indicate that there were significant differences between the two treatments of the genes at *p* < 0.05.

Now that we found that the BV nanomaterial can change root length and lateral root number, some genes related to root development were also further investigated. Therefore, we selected three types of genes, among which two genes mainly affected the primary roots (*ADC1*, *DAR2*), three genes mainly affected the lateral root (*ARF19*, *CKX1,* and *ERF6*), and one gene affected both (*IQM3*) (Figure 9). Surprisingly, in the primary roots, *ADC1*, *DAR2,* and *IQM3* showed the same trend. After treatment with BV, the expression levels of the three genes were significantly upregulated. These results suggest that BV may be involved in the regulation of plant taproot development in a variety of ways related to hormones and polyamines.

Furthermore, in the lateral root, the related genes show a more complex phenomenon. *ARF19*, as a gene-mediating hormone regulator of lateral roots, is the most in-depth and clear regulatory pathway (Okushima et al., 2005). In the experiment, BV treatment significantly increased the expression of *ARF19*. *CKX1* could mediate cytokinins to regulate lateral root development, which was significantly downregulated after BV treatment. Moreover, *IQM3* showed a similar level between the BV treatment and the control. *ERF6* was significantly upregulated in roots treated with BV. All of these results showed that BV can regulate root development by mediating hormones.

## Discussion

BV is a new type of environmental protection material, and its excellent performance in many fields has been verified (Castiglione et al., 2011). With the widespread use of BV, it will inevitably flow through the environment. BV is an environmentally friendly and low-carbon metal oxidation material (Yin et al., 2010). In this study, considering that BV may be first enriched in plants, we selected the model plant *Arabidopsis* as the research object to investigate the effects of BV on plants in terms of many aspects.

We first observed the root changes after BV treatment. Different from most nanomaterials that exert toxicity against plants (Dimkpa et al., 2012; Zou et al., 2016; Yang et al., 2018), root development following treatment with different concentrations of BV showed an opposite phenomenon (Figures 2,3). We found that root development was promoted by adding an appropriate concentration of BV, while other concentrations inhibited root development. This is similar to some plant growth regulators and indicates a dual nature. This is not the first observation of the phenomenon of dual effects on plants, and a report showed that GO exhibited a similar phenomenon (Park et al., 2020). There may also be another explanation, that is, the dual effects are related to the concentration of BV. This hypothesis is based on the fact that BV is not detected on the surface and different parts (root rip, exptension zone, and mature zone) of the treated roots observed by EDS (Figures 6C–E and Supplementary Figure S1). This implies that BV plays a role after entering the cells. In contrast to animal cells, plant cells have cell walls and almost no phagocytosis (Tenhaken et al., 2015). Before entering plant cells, nanomaterials must penetrate cell walls and plasma membranes. When the concentration of BV is low, it cannot be well-absorbed by plant roots, so it cannot play a role. When the concentration reaches a certain value, BV can be absorbed by the roots and act. The effect of nanoparticles on cucumber involves a similar mechanism (Zhang et al., 2011). The XRD results also proved this point (Figures 6A,B). However, we need to pay attention to the fact that BV is not enriched in the root but in the leaf. It is likely that BV is absorbed by roots and transported to leaves. A similar phenomenon occurs when nanoplastics of different charges accumulate in *Arabidopsis* (Sun et al., 2020). Although there was no enrichment of BV in roots, a small amount of BV was enough to have a significant effect. Further studies found that BV mainly promoted the development of elongated regions, but had limited effects on other regions. The results of SEM also proved this point (Figure 4). Interestingly, BV not only has dual effects on the development of taproots but also on the development of lateral roots. Previous studies have shown that the development of lateral roots can be affected by many kinds of plant hormones (Zhao et al., 2014). In our study, the effect of BV nanomaterials on lateral roots may be achieved by changing plant hormones. The lateral root is connected to the taproot, and water and nutrients can flow through the catheter and sieve tube (Casimiro et al., 2001). Generally, the growth of lateral roots was inhibited by the growth of taproots, especially near the root tip (Casimiro et al., 2001). To a certain extent, BV treatment was in line with this trend. It is necessary to further study these phenomena, and the cause of these different modes of influence can increase our understanding of how BV affects plants.

Considering that BV can affect the growth of roots, we also observed the leaves (Figure 5). Not surprisingly, we found that the leaves also changed significantly after BV treatment. The most obvious change was that the number and size of stomata expanded. The stomata is an important gas exchange channel between the leaves and air. The regulation of stomatal opening and closing plays an important role in transpiration, photosynthesis, and other important biological processes (Davies et al., 1991). Under drought conditions, plants reduce stomatal opening and closing or even close stomata to reduce transpiration intensity, to reduce water loss to adapt to drought environments (Davies et al., 1991). In addition, stomatal closure can also affect the absorption of CO_2_ by plants and directly affects the intensity of photosynthesis (Bonan et al., 2008). This means that BV treatment can promote the development of *Arabidopsis* by affecting stomatal-mediated photosynthesis and transpiration.

In view of the fact that BV can promote the development of *Arabidopsis,* we further studied the related physiologic indicators. Subsequently, TTC and NBT staining was performed to observe the root activity and production of ROS in seedlings. When organisms are stressed, the production of ROS in the body will increase greatly and when it exceeds the antioxidant defence capacity of organisms, cellular components such as lipids, proteins and nucleic acids will be irreversibly damaged under the action of O_2_^.−^ to interfere with cell metabolism or cause cell death (Williams et al., 2014). Succinic acid is a key intermediate in the tricarboxylic acid cycle. Under physiologic conditions, succinate dehydrogenase in mitochondria oxidizes succinate to fumaric acid and releases electrons to participate in oxidative phosphorylation (Chouchani et al., 2014). The reduction in TTC indicates that the activity of the dehydrogenase can be used as an indicator of root activity. As a method to identify root vitality, TTC staining is based on the principle that living tissues can produce hydrogen ions under the action of the dehydrogenase and has a reduction ability. The depth of tissue coloration indicates the strength of root activity. After growing in the half-strength MS medium for 6 days, we found that the root tip color of 200 μg ml^−1^ BV was significantly darker than that of the control, indicating that the seedlings treated with appropriate concentrations of nanomaterials increased the root activity of *Arabidopsis* seedlings. Xie et al. (2019) also reported that plant root activity and plant root growth were positively related under GO treatment in napus seedlings. This suggests that BV can affect the tricarboxylic acid cycle and plays a positive role in enhancing plant activity. Superoxide dismutase (SOD) is an enzyme that scavenges O_2_^.−^ (Smith et al., 2003). O_2_^.−^ can reduce NBT to blue methylhydrazone, so SOD inhibits the formation of methylhydrazone. The deeper the blue color of the tissue treated with the NBT solution, the lower the enzyme activity. In a reverse situation, the enzyme activity is higher. Therefore, this method can be used to measure O_2_^.−^. In this experiment, we found that *Arabidopsis* seedlings at 200 μg ml^−1^ BV were slightly lighter than those of the control, indicating that the accumulation of O_2_^.−^ in seedlings was reduced by an appropriate concentration of BV. Plants respond to nanoparticle-mediated stresses through ROS generation (Begum et al., 2012). It is widely accepted that the toxicity of nanoparticles on plants is commonly evident at high concentrations and attributed to the generation of reactive oxygen species (ROS) (Marslin et al., 2017). However, a report showed that SOD activity was widely stimulated after the exposure of plants to TiO_2_NPs and reduced the level of total ROS. Our data are consistent with the results (Melo et al., 2021). This result suggested that BV enhanced the SOD activity and promoted plant growth by reducing the accumulation of ROS. In addition, we also studied the antibacterial activity of BV. The results showed that BV had an obvious inhibitory effect on some pathogens causing crop diseases. This also provides a new potential means for crop disease control. Interestingly, this result can also be used to explain why low concentrations inhibit root development and high concentrations promote root development. Based on the bacteriostatic effect of BV, when it cannot be absorbed by roots, it may inhibit the development of roots when it is free outside the roots.

Finally, we quantitatively studied some genes related to root development to analyze the molecular mechanism by which BV affects root development. *ADC1* can affect the synthesis of polyamines in *Arabidopsis* (Maruri-López et al., 2017), and studies have shown that polyamines can regulate root length (Gurung et al., 2012). *DAR2* can be associated with a variety of hormones and then affect the development of the root meristem (Peng et al., 2013). *ERF6*, as a transcription factor, is also regulated by hormones, thus affecting the development of roots (Eysholdt-Derzsó et al., 2017). In *Arabidopsis*, the *IQM* family belongs to the calmodulin-binding protein family with an IQ motif. *IQM3* is involved in the regulation of plant root development (Xu et al., 2019). In our study, BV treatment resulted in significant changes in these genes, which can regulate the development of taproots. In the lateral roots, the related genes showed more complex phenomena. In general, BV can affect the expression of plant hormone–related genes and may regulate root development through hormone-mediated pathways.

## Conclusion

In our study, BV promoted root development in *Arabidopsis* by increasing the length of taproots and the number of lateral roots. At the same time, BV also increases the number and size of stomata. The results of tissue staining showed that BV played a positive role and enhanced plant vitality. Moreover, BV had an inhibitory effect on some pathogens causing crop diseases. In addition, the expression levels of root development–related genes changed. In conclusion, proper concentrations of BV are expected to be used as promoters for plant growth and development. BV is expected to be widely used in plant-related fields due to its excellent properties, low production cost, and antimicrobial properties. However, there are many problems to be solved. For example, only *Arabidopsis* was used as an experimental material in this research, whereas how BV affects other plants remains to be explored. Finally, it is necessary to explore the mechanisms by which nanomaterials affect plants and deepen the understanding of these mechanisms.

## Data Availability

The original contributions presented in the study are included in the article/Supplementary Material; further inquiries can be directed to the corresponding author.
