# Supplementary material for: Bismuth Vanadium Oxide Can Promote Growth and Activity in Arabidopsis thaliana
Source: Front Chem. 2021 Nov 11;9:766078. doi: 10.3389/fchem.2021.766078 (PMC8632446; doi:10.3389/fchem.2021.766078)
Supplement: Supplementary file 1 [file Table1.docx]

**Supplementary Table S1 Primers used for qRT-PCR**

| Gene name | Locus | Direction | Primer sequence | Length (bp) |
| --- | --- | --- | --- | --- |
| ERF6 | *AT4G17490* | forward | TTGTAGCAGCAGAGGAGAAGAG | 106 |
|  |  | reverse | CCAAACACGAGTTCCACGAC |  |
| ARF19 | *AT1G19220* | forward | TCCAGTGCTGCAATCAGTTC | 112 |
|  |  | reverse | CCTCCACCATTCATGATTCC |  |
| CKX1 | *AT2G41510* | forward | ACAGAGGAAACAAGCCTACGAC | 102 |
|  |  | reverse | TGACTTTGCGAGTTGGATGG |  |
| ADC1 | *AT2G16500* | forward | TGTGGCTTCGGTTAGGTTTG | 138 |
|  |  | reverse | GTCTCATGTTGTTGACCAGCTG |  |
| DAR2 | *AT2G39830* | forward | AGCATGAGTTCTCTCTGTCAGG | 142 |
|  |  | reverse | CCAAAACGGATGGCATCGATAC |  |
| IQM3 | *AT3G52870* | forward | GGAGGGTGATTGTTGACAATGG | 120 |
|  |  | reverse | AGGTTCACTGCGTTCTCTCTG |  |
| ACT2 | *AT3G18780* | forward | GCCATCCAAGCTGTTCTCTC | 270 |
|  |  | reverse | GCTCGTAGTCAACAGCAACAA |  |
