# Supplementary material for: Bismuth Vanadium Oxide Can Promote Growth and Activity in Arabidopsis thaliana
Source: Front Chem. 2021 Nov 11;9:766078. doi: 10.3389/fchem.2021.766078 (PMC8632446; doi:10.3389/fchem.2021.766078)
Supplement: Supplementary file 2 [file Image1.pdf]

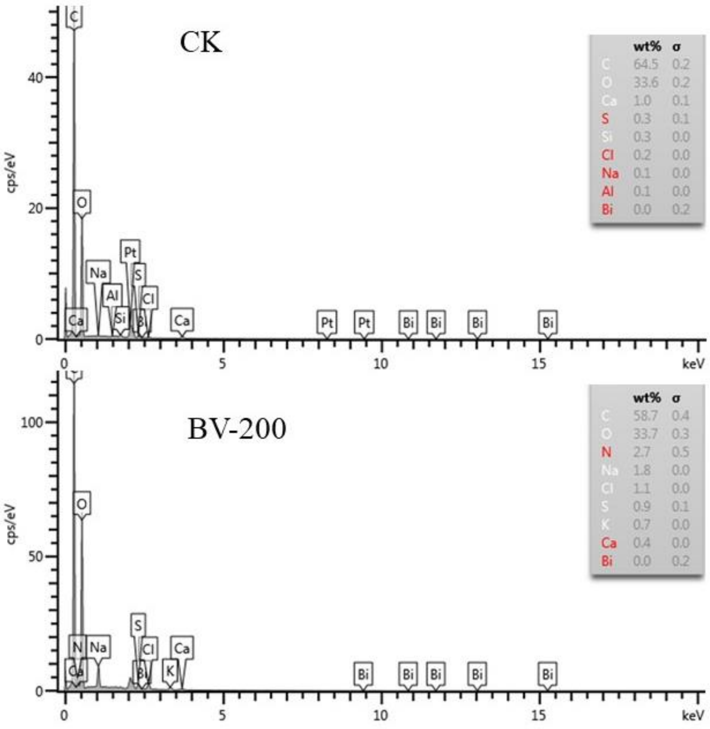

Supplementary Figure S1 Effect of nanomaterial BV on the extension zone of taproot in *Arabidopsis*.

CK: Without nanomaterial BV treatment. BV-200 was treated with BV about 200 μg mL^{-1}. N=3.
